# Patients’ willingness to attend the NHS cardiovascular health checks in primary care: a qualitative interview study

**DOI:** 10.1186/s12875-015-0244-7

**Published:** 2015-03-08

**Authors:** Caroline E Jenkinson, Anthea Asprey, Christopher E Clark, Suzanne H Richards

**Affiliations:** Primary Care Research Group, University of Exeter Medical School, Smeall Building, St Luke’s Campus, Exeter, EX1 2 LU UK

**Keywords:** Cardiovascular health check, Uptake, Barriers, Motivators, Patient qualitative interviews, Primary care

## Abstract

**Background:**

The NHS Cardiovascular Health Check (NHSHC) programme was introduced in England in 2009 to reduce cardiovascular disease mortality and morbidity for all patients aged 40 to 74 years old. Programme cost-effectiveness was based on an assumed uptake of 75% but current estimates of uptake in primary care are less than 50%. The purpose of this study was to identify factors influencing patients’ willingness to attend an NHSHC. For those who attended, their views, experiences and their future willingness to engage in the programme were explored.

**Method:**

Telephone or face-to-face interviews were conducted with patients who had recently been invited for an NHSHC by a letter from four general practices in Torbay, England. Patients were purposefully sampled (by gender, age, attendance status). Interviews were audio recorded, transcribed verbatim and analysed thematically.

**Results:**

17 attendees and 10 non-attendees were interviewed. Patients who attended an NHSHC viewed it as worthwhile. Proactive attitudes towards their health, a desire to prevent disease before they developed symptoms, and a willingness to accept screening and health check invitations motivated many individuals to attend. Non-attendees cited not seeing the NHSHC as a priority, or how it differed from regular monitoring already received for other conditions as barriers to attendance. Some non-attendees actively avoided GP practices when feeling well, while others did not want to waste health professionals’ time. Misunderstandings of what the NHSHC involved and negative views of what the likely outcome might be were common.

**Conclusion:**

While a minority of non-attendees simply had made an informed choice not to have an NHSHC, improving the clarity and brevity of invitational materials, better advertising, and simple administrative interventions such as sending reminder letters, have considerable potential to improve NHSHC uptake.

**Electronic supplementary material:**

The online version of this article (doi:10.1186/s12875-015-0244-7) contains supplementary material, which is available to authorized users.

## Background

In April 2009, the Department of Health launched the NHS Cardiovascular Health Check (NHSHC) programme with the aim of reducing cardiovascular disease (CVD) mortality and morbidity in England [1]. This primary prevention programme invites adults (aged 40-74 years) for an assessment if they have not previously been identified as at high risk (20% or higher) of developing cardiovascular disease within ten years, and are not being treated for a range of CVD conditions [2] including: coronary heart disease; chronic kidney disease (CKD) (classified as stage 3, 4 or 5 within NICE CG 73); diabetes; hypertension; atrial fibrillation; transient ischaemic attack; hypercholesterolaemia; heart failure; peripheral arterial disease; or stroke. Patients who are prescribed statins for the purpose of lowering cholesterol are also ineligible for a NHSHC. The NHSHC measures body mass index, blood pressure, renal function, glycosylated haemoglobin and cholesterol, and captures information on family history of CVD and relevant lifestyle risk factors. Dementia awareness (65-74 year olds only) and alcohol use were added in 2013. Individuals identified at high risk (>20%) of developing CVD within the next ten years, based on a QRISK2 score [3], are offered clinical management, such as prescribing and lifestyle interventions. Where findings suggest a new diagnosis of hypertension, chronic kidney disease or diabetes, participants enter the appropriate diagnostic pathway and, where confirmed, are entered to the appropriate primary care disease registers.

NHSHC uptake was introduced as a performance indicator within the Public Health Outcomes Framework from April 2013 [4]. Local authorities should annually offer 20% of their eligible population an NHSHC, inviting all eligible patients within a five year cycle [5]. High uptake is critical to the programme’s success; economic modelling exploring its cost-effectiveness was based on a 75% uptake rate [6], yet recent data indicated much lower rates (between 20 and 50%) [5,7-11]. Whilst staff experiences of delivering NHSHCs have been reported patients’ views have not [12]. Patient views towards cardiovascular screening have been explored in populations at high risk of developing CVD, but these may differ from people at lower risk [13].

The Check-Up Study consisted of two stages: a quantitative analysis of patient datasets (Stage 1), and patient interviews (Stage 2). We report here stage 2; this interview study explored factors influencing patients’ willingness to attend an NHSHC, their views and experiences of the NHSHC and future willingness to engage with the programme.

## Method

### Recruitment and data collection

The study was ethically approved by the NRES Committee South West - Cornwall & Plymouth, Bristol Research Ethics Committee Centre, Level 3 , Block B, Whitefriars, Lewins Mead, Bristol BS1 2NT (12/SW/0314).

In Devon, until April 2011, only Torbay and Southern Devon Health and Care NHS Trust had piloted and commissioned a fully embedded model of the NHSHC (in 18 of its 20 general practices). Practices used various invitation methods to invite eligible patients to have an NHSHC, ranging from opportunistic invitations during GP consultations to systematic mail shots. Only practices systematically inviting patients by letter were eligible for this study since patient medical records were used to identify patients’ NHSHC attendance status. Thirty interviews were planned, with ten patients who had attended their NHSHC (attendees) and twenty patients who had chosen not to attend (non-attendees). To help understand how to improve the uptake of NHSHCs, more non-attendees were invited to participate in the interview study.

In May 2013, practices identified all individuals sent an NHSHC invitation letter in the last three months. By definition eligible participants were free of chronic disease; therefore there were no pre-specified exclusion criteria. However, the list was screened by a clinician to exclude anyone for whom an interview was deemed inappropriate (for example, patient recently bereaved). The list was divided into attendees and non-attendees; the latter defined as those who had failed to respond to their NHSHC invitation within four weeks. Practices were asked to randomly sample individuals from their lists of attendees and non-attendees using Microsoft Excel software, to each achieve a maximum sample of 20 attendees and 80 non attendees stratified by gender and age (either 40-65 years or 66-74 years). These age bands were an attempt to recruit people of different working status.

Practices sent patients a recruitment pack containing a patient letter, information sheet, reply slip and pre-paid envelope. Respondents returned their contact details on the reply slip directly to the Associate Research Fellows (CJ and AA), who then contacted them to discuss the study and arrange an interview. To maximise recruitment, CJ and AA conducted the interviews either over the telephone, or face-to-face in the patient’s home. Both CJ and AA are experienced university, qualitative researchers independent of the NHSHC delivery teams. The consent form was discussed and was either posted (telephone interviews), or completed prior to the face-to-face interviews. Telephone interviews were only analysed on the receipt of the signed consent form. Semi-structured interviews were conducted using a topic guide developed following discussions among the researchers (CJ, AA and SR) and a literature search.

All patients were asked general questions about themselves (age, working status, general health) and their awareness of NHSHCs. The topic guide included:

- Do you remember being invited for a health check by your local GP surgery?
- Can you remember how were you invited?
- Did you attend the offered check?

Attendees were also asked:

- What prompted you to take up the health check?
- How did you feel beforehand?
- Can you briefly describe what happened during your health check?
- Did you receive any results or feedback from your health check?
- Were you told your level of cardiovascular risk?
- If reporting being at high risk, participants were asked: what treatment was offered? (medication, lifestyle advice), how did you feel about this and were they referred to see another health professional?
- If you had another invitation for a health check, what would encourage you to attend one in the future?
- What would help – is there anything we could do to make it easier?
- To what extent has the health check motivated you to change your health behaviour?
- What would help you to plan the changes? Any barriers to change?
- Have you made any changes?

Non-attendees, who were aware of the health check, and chose not to attend, were asked:

- What do you think stopped you from having the health check?
- What would encourage you to attend a health check in the future?
- What would help – is there anything we could do to make it easier?

Our approach ensured the same topics were covered in each interview while allowing for flexibility and probing with individual interviewees. Furthermore, this approach also enabled the exploration of the interviewee’s own experiences, meanings and values rather than imposing preconceived assumptions. The interviews were audio recorded and transcribed verbatim by AA; transcripts were fully anonymised. Field notes were recorded by CJ and AA during and after the interviews.

### Data analysis

Interview transcripts were analysed using QSR NVivo (version 10) by CJ. Codes were developed iteratively: transcripts were continually revisited in the light of subsequent transcripts to ensure that codes were comprehensively applied [14]. Blind double coding was undertaken by AA on five transcripts. Discussions among the researchers (CJ and AA) verified that codes were applied systematically, accurately and appropriately. Some codes arose directly from answers to interview questions, however, an inductive approach was used to identify concepts emerging directly from the data [15]. To identify the salient themes and concepts emerging, data were analysed thematically [16-18]. This study was undertaken and reported in accordance with COREQ guidance [19] (see Additional file 1).

## Results

### Sample characteristics

All eighteen practices were invited to take part in stage 1, however only six practices were able to generate appropriate datasets. All six practices agreed to take part in stage 2, however only the four that employed systematic invitation methods were eligible. A total of 334 individuals (74 attendees, 260 non-attendees) were invited to take part; 27 (8%) responded and were interviewed between June and August 2013 (Table 1). Three patients originally identified as non-attendees had attended their NHSHC by the time of interview, and were therefore re-classified as attendees. Two practices had issued NHSHC invitations to fewer than the maximum intended sample size at the time of study invitation, so no sampling was undertaken and all eligible patients from those practices were invited to participate. Interviews were relatively short, lasting no more than 30 minutes. Table 2 describes the demographics of interviewees (n = 27; 17 attendees, 10 non-attendees).

**Table 1:** **Response of patients invited to take part in the study**

	**Number of patients who responded to study invitation**				
	**Practice A**	**Practice B**	**Practice C**	**Practice D**	**All**
**Attendee** (total invitations sent)	3 (20)	1 (14)	9 (20)	1(20)	14 (74)
**Non-attendee** (total invitations sent)	2 (80)	3 (25)	5^a^ (80)	3 (75)	13^a^ (260)

^a^Three of these five non-attendees subsequently attended for NHSHCs so for the interview and analysis were reclassified as attendees.

**Table 2:** **Characteristics of the interviewees**

	**Attendees (n = 17)**		**Non-attendees (n = 10)**	
	**Female (n = 12)**	**Male (n = 5)**	**Female (n = 6)**	**Male (n = 4)**
**Age**				
40-65 years	3	3	5	3
66-74 years	9	2	1	1
**Working status**				
Employed	4	2	3	1
Unemployed	0	1	1	0
Retired	8	2	2	3
**Living arrangements**				
With partner/spouse	12	4	6	4
Alone	0	1	0	0
**Living with dependents**				
Yes	1	1	0	2
No	11	4	6	2

Attitudes towards general health were comparable between attendees and non-attendees. A similar range of lifestyle behaviours, such as activity levels and smoking status, was reported by participants from both groups. Most interviewees reported enjoying good health, with many holding fatalistic views towards developing future ill health.

*Patient (P): ‘No, I think as long as I look after my diet and keep a mindful eye.’*

*Interviewer (I): ‘It’s not something that you dwell on?’*

*P: ‘No, there’s no point, really, is there, if it’s going to happen it’s going to happen.’* (Attendee 10, female, retired, 68 years)

*‘I always say, you know, if you get something, you get something, and I know you can have these check-ups but I’ve had my brush with [death], you know … and I just live life now, to the full, and just enjoy it.’* (Non-attendee 7, male, retired, 43 years)

### Main findings

Findings concerned factors influencing NHSHC uptake, patients’ NHSHC experiences, future willingness to engage in the programme and suggestions to improve NHSHCs.

#### Factors influencing uptake

Possible factors influencing individuals’ choices regarding NHSHC uptake were grouped according motivators and barriers to attendance (Table 3).

**Table 3:** **Possible factors influencing patients’ choices when considering an NHSHC**

**Motivators**	**Barriers**
**MOT** ^**a**^	**Not a priority**
better to find out	too busy
reassurance	perceives healthy lifestyle/no health worries
aware health can deteriorate with age	avoids thinking about health
**Proactive towards health**	**Recent monitoring**
happy to receive advice	other conditions regularly monitored
chance to change lifestyle	blood pressure/cholesterol recently checked
prevention better than cure	
**Other health problems**	**Misunderstanding NHSHC**
not feeling well	did not receive invitation
get treatment/results for other conditions	misread letter/information sheet
recommended by a health professional	only available for limited time
**NHS provisions**	**Negative view of NHSHC**
accept everything offered	bad experience of someone else
must be a reason for invitation	fear of outcome
	being told off or mollycoddled
	**Relationship with GP practice**
	perceived wasting Dr/nurse’s time
	difficulty arranging appointment
	good not to go to GPs

^**a**^MOT refers to the annual test legally required in the UK to check that a car conforms to regulations governing safety and emissions.

##### Motivators to attend

Many interviewees viewed the NHSHC as the human equivalent of the ‘MOT test’ (annual test required by law to ensure cars conform to safety and emissions regulations), that is, to identify and address any potential health problems. Patients welcomed both the opportunity the NHSHC affords for prevention and early treatment of underlying conditions, and the chance to be aware of their health issues, adopt healthier lifestyle habits, and act on professional advice.

*‘I just think it makes you more aware and if you do have any problems you have the chance to actually, you know, be proactive to it rather than reactive when maybe things are a bit too late.’* (Attendee 1, female, employed, 44 years)

Some were motivated by their own experience of long-term conditions (unrelated to CVD) and attended solely because they felt unwell at the time of the invitation, or combined the NHSHC appointment with receiving results or treatments for ongoing ailments. The fact that health inevitably deteriorates with age was another motivating factor.

*‘Well, I mean, if it’s caught early and it can be dealt with, you’ve got a much better chance of surviving a heart attack or a stroke or whatever.’* (Attendee 7, female, retired, 73 years)

*‘…I’d go for years without seeing a doctor. And unfortunately when you reach the magic 50, um, things start going wrong, and er, sometimes they go pretty quickly as well…’* (Attendee 13, male, employed, 62 years)

The final motivator concerned patients’ attitudes towards the NHS: many attendees believed it important that they should accept everything that was offered.

*‘Yes, it’s like you get your letter to go and have your mammogram, it’s part of it, take all the help you can get!’* (Attendee 11, female, retired, 73 years)

*‘Well yeah, it’s just the fact it’s on offer that’s why I said “Yes please.”’* (Attendee 4, female, employed, 52 years)

However, one attendee thought there must be a negative health reason not known to him for being invited.

*‘Um, I was surprised they picked me, there must have been a reason why they picked me, I’m not sure why I’d been asked, but I thought “Well, they sent that for a reason so I’d best go and have it done."’* (Attendee 12, male, employed, 52 years)

##### Barriers to attendance

Reasons for non-attendance ranged from patients being either too busy or feeling healthy enough not to need a check, to others wishing to avoid thinking about their own health. Some felt the NHSHC was unnecessary due to their receiving regular monitoring for other health conditions, or having their blood pressure or cholesterol recently checked. A general misunderstanding of the aim of NHSHCs was a key factor contributing to non-attendance. Both invitation letters and information leaflets were sometimes incorrectly read leading to confusion about the range of health measures assessed.

*‘Yes, um, I didn’t realise that it was dementia…And I certainly didn’t know that it was, um, diabetes and kidney, I thought it was purely cholesterol.’* (Non-attendee 2, female, retired, 67 years)

*‘I sort of vaguely read over it, it was about 3 pages long and to be honest I was bored after the first page.’* (Non-attendee 1, female, employed, 50 years)

Negative views from friends influenced some interviewees; while others stated that they disliked being mollycoddled by health professionals, disliked being given lifestyle advice, or feared being told they had a disease.

*‘Yes, but I do know a lot of my friends had the same letter and they thought one, they’re overweight like me and probably drank too much alcohol and when they said we’re having a liver test, and “I’m not going, I’m not going”.’* (Attendee 8, female, retired, 73 years)

*‘[laughs] It’s funny actually, ’cause that was one of the things that went through my mind, I said to [name of husband], I said “Well I’m going to try and lose two or three pounds before I go, because I’m sure they’ll tell me I’m overweight”.’* (Non-attendee 4, female, employed, 57 years)

Finally, some non-attendees did not wish to waste health professionals’ time, while others found it difficult arranging an appointment when they were ill so doubted they could obtain an appointment when feeling well. Sometimes specific health experiences constituted a disincentive to attendance.

*‘And when I was, went through my really bad period I just seemed to be, you know, in and out of the doctors and various other places so much and I just, I love the fact now that I don’t go near the place!’* (Non-attendee 5, male, employed, 61 years)

All interviewees thought NHSHCs were a good idea except two interviewees, one of whom was a 66 year old female. Although an attendee herself, her comment highlighted a potential barrier.

*‘…I am of the generation where you didn’t go to the doctor unless there was something wrong with you, you didn’t waste the doctor’s time…To be quite honest, when I first heard about this, I thought “Well, that’s a waste of money, they’ve got to pay all those nurses and that, when they should be in the hospital!”…Because if someone knows there’s summat wrong with them, they go to the doctor and if they’re quite happy jogging along, then let ’em! [laughs]’* (Attendee 17, female, retired, 66 years)

#### NHSHC experience

All but one interviewee remembered receiving the invitation from their general practice to have an NHSHC. Attendees recalled varying amounts of detail about their NHSHC, but usually remembered it as a pleasant experience.

*‘Well, she did my height, my weight, my blood pressure, she took some bloods, and um, just asked me generally about what sort of diet I had, what exercise I had, whether I smoked, how much I drank.’* (Attendee 5, female, employed, 48 years)

*‘Um, to tell you truth I can’t really remember an awful lot of it because it was absolutely fine, just sailed through, um, the nurse was very pleasant, um, I think overall it is a very, very good idea.’* (Attendee 16, female, retired, 69 years)

Many interviewees reported receiving simple lifestyle advice which was met with varying degrees of eagerness to heed. Some attendees actively tried to follow the advice given. One patient, for example, was referred to a smoking cessation clinic and another was referred for exercise.

*‘Yeah, I go to, um, the doctor’s once a fortnight and I see a smoking lady down there, and um, we talk it through and bring me to a machine just to check I haven’t been smoking…I’m on 25 milligrams [nicotine patches], which is high, at the moment, for so many weeks, I think it’s between eight and ten weeks. Then I think the dose goes down to fifteen milligrams, and the last one is a five or a ten milligram one. And that’s it then, they just wean you off…I’ll definitely give up the cigarettes, and take more exercise, drink plenty of fluids, water-wise and just try to not worry so much about things that’s going on around me.’* (Attendee 12, male, employed, 52 years)

*‘…I asked if I could be referred to a gym…and they said “Yeah”, I could join the one at [name of area], which is what I did…So I’ve been going twice a week for an hour.*’ (Attendee 7, female, retired, 73 years)

*‘Er, I just watch the amount of um, butter and fat levels I have that might affect my cholesterol, yeah, just become a little bit aware of that, which I wasn’t before. That*’*s about it.’* (Attendee 14, male, retired, 66 years)

However, some attendees had no intention of following the recommended changes to lifestyle.

*‘Yes, they give me loads of advice. Lose weight, stop drinking, to change me diet, both of which I’ve ignored!’* (Attendee 3, male, unemployed, 62 years)

Around half of the attendees remembered being given their CVD risk score. Although there were no reported CVD diagnoses resulting from the NHSHC attendees interviewed, one person was diagnosed with anaemia and another was identified as having high blood pressure, of which she was informed during the NHSHC and subsequently was followed up to confirm a new diagnosis of hypertension.

*P: ‘Because when she did the blood pressure she kept saying “Were you worried about coming” because it was high. And I said “No, I’m not, you know, no!”’*

*I: ‘Laid back!’*

*P: ‘Yeah, and in actual fact she went through it all when I went two weeks later, ’cause I had to go back, I think I went 3 times, every 2 weeks, I think it was, not 4 weeks, for the blood pressure, just to make sure before they put you on tablets, yes.’* (Attendee 8, female, retired, 73 years)

#### Engagement in future NHSHCs

All attendees were enthusiastic about having a future NHSHC. Furthermore, all but two non-attendees said they would now attend if they were offered one again, largely as a result of the interviewer explaining the purpose of the NHSHC, or being able to answer any questions that the interviewee had about the health check.

*‘I think, well, I eat healthily, you know, and I believe in a healthy lifestyle. I know there are other internal things which could go wrong. But yeah, I would definitely have it again if it came up next week, you know, I didn’t realise it was all about different things.’* (Non-attendee 7, male, retired, 43 years)

*‘…I consider myself foolish not to have gone, because there’s me thinking, oh, you know, I can walk this far and um, you know, and I’m fit and healthy and I sleep well…but in retrospect, unless it was in bold print, what they were checking for, and I’m not thick, you know, but obviously this [details from NHSHC invitation] didn’t register.’* (Non-attendee 2, female, retired, 67 years)

#### Recommendations for improvements to the NHSHC

Suggestions to increase the NHSHC uptake fell into two broad categories: better promotion and improvements to its administration. Many interviewees believed promotion could be improved, suggesting both positive and negative reinforcement. Ideas ranged from advertising on the television, in libraries or school newsletters, to celebrity endorsement and NHSHCs being written into scripts of television programmes. Eye catching posters displayed in GP waiting rooms and chemists were also suggested.

*‘Yeah, you could do. Um, television advert? …Bring out the worst scenario, you know. “If I hadn’t gone for this check, I would not have found this out.”’* (Attendee 8, female, retired, 73 years)

*‘Um, saying, you know, “No one likes to get old, but, you know, it happens”, even famous people have ill health as they get older. And if you have a couple of fit, forty plus footballers on the TV or something, or actresses, or singers, something like that, you know…’* (Non-attendee 4, female, employed, 57 years)

Several interviewees suggested simplifying the invitation letter and information sheet, and highlighting key aims using bold text. Some suggested that practices should send reminder letters to emphasise the importance of attending, while others suggested early morning or evening appointment slots to accommodate working patients.

## Discussion

### Summary

Numerous factors influencing patients’ decisions surrounding NHSHC attendance or non-attendance were identified. Attendees tended to be proactive towards their health and willingly accepted invitations for preventative checks or screening. The NHSHC was often viewed as reassuring; providing a valuable opportunity to be checked for otherwise hidden health problems. Non-attendees reported various barriers, with many simply not viewing it as a priority. Misunderstandings regarding its purpose were common, particularly among those being monitoring for other, unrelated health conditions, or who had their blood pressure or cholesterol recently measured. Fear of what results might be revealed, or of being ‘told-off’ during the appointment deterred others. In contrast, some non-attendees viewed it as a waste of health professionals’ time, or had made an informed choice and preferred not to use the practice when they were in good health. Participants’ suggestions to encourage NHSHC attendance included better advertising and promotion, improving administration and clarifying invitation materials.

### Strengths and limitations

Patients who disengage with health services are also difficult to recruit in research. In anticipation of this problem, four non-attendees were invited to take part for each attendee invited. Far fewer non-attendees than attendees responded; ten were successfully interviewed, but this was below target and data saturation may not have been achieved. Nevertheless both men and women from diverse socio-economic backgrounds, including unemployed, retired and manual or professional workers were successfully interviewed. Ethnic minority populations in South Devon are small and our findings may not represent the experiences of people from different ethnic groups. Similarly, interviews were only conducted in English so the potential for language being cited as a barrier to having an NHSHC could not be explored. There was no evidence that attendees and non-attendees differed in their distribution of lifestyle risk factors or existing health conditions, however our sample was too small to allow that sort of comparison or indeed to explore gender differences. Furthermore, barriers cited by participants, particularly those pertaining to the relationship with the GP practice, may be specific to the four practices involved in the study. In addition, practice personnel were confident in their ability to randomly select a sample from each subset, so no further guidance or monitoring were provided or undertaken. For pragmatic reasons such as identifying non-attendees, practices employing opportunistic NHSHC invitation methods were excluded from the current study. By restricting our sampling to practices employing systematic methods to invite patients for NHSHCs, our findings may be not be representative of patients who elect not to attend from practices inviting such individuals solely through opportunistic methods. Future studies should target greater numbers of practices covering different geographical regions and ethnic minorities, to permit exploration of the role that the practice itself plays in promoting or impeding patient participation in health checks and screening. In addition, the role of the GP as a motivator or barrier could be explored in depth.

### Comparison to existing literature

Three quantitative studies, located in deprived inner city areas of London [8,9] and Birmingham [10] identified patient and practice factors independently associated with NHSHC uptake. Older people (65-74 year olds) [8-10] and patients of south Asian backgrounds [9,10] or mixed ethnicity [9] were more likely to attend than younger people or those from a white background. Also, people recorded as ‘smokers’ [10], or individuals registered with small practices [9,10] were less likely to attend. These demographic differences have also been observed in our own ongoing analysis of uptake in a rural practice [20]. While these studies identified patient groups less likely to attend an NHSHC, unlike our qualitative study, the reasons underlying decision making were not explored.

Several previous qualitative studies have explored patient experiences of attending cardiovascular screening [21-23], and of their response to being found to be at cardiovascular high risk and the treatment options made available to them [13,22]. Only one qualitative study [24], conducted in four practices in a socially deprived area of South London, interviewed a mix of patients who did (n = 17) or did not attend (n = 10) for an NHSHC. Consistent with our data, this study found that the NHSHC programme needed to raise public awareness to ensure that people are better informed about its purpose and to tackle misconceptions, and that non-attenders may fail to prioritise the health check in the context of their busy lives or through a desire to avoid unwelcome advice to change their lifestyle.

Other research identifying barriers to engaging with preventative interventions focus on patients not acknowledging their risk of developing disorders, and poor communication about the services offered [25]. An evidence synthesis, commissioned to support NHSHC implementation, identified a paucity of research into factors influencing a patients’ willingness to engage with cardiovascular screening [26], and instead drew on the extensive literature from cancer screening programmes. There is robust evidence that client reminders, tailored information sheets, one-to-one education, and provider audit and feedback mechanisms are effective at increasing cancer screening uptake [27]. Our participants endorsed the need for clearer NHSHC invitation materials, for practices to routinely use reminder letters, and for better promotion using mass media [25].

While non-attendees failed to prioritise the NHSHC, or simply misunderstood what it involved, this may also be partly influenced by some health professionals’ reluctance to actively endorse or promote it. Two recent systematic reviews of interventions providing primary prevention of coronary heart disease found no proven benefits in risk reduction [28,29]. The NHSHC programme challenged these findings with recent English data that suggested patients may sustain a reduction in their CVD risk scores one year following their NHSHC [30]. This lack of clarity has resulted in highly publicised scepticism of the NHSHC programme in some primary care circles [31-33]. At present, while acknowledging the absence of a robust evidence base, the Department of Health has re-iterated that NHSHCs are a public health priority, but should be accompanied by ongoing research and evaluation [34]. The supporting evidence base must be improved if health professionals are to actively encourage patients to attend for an NHSHC. However, while many non-attendees might be persuaded to attend, a minority had made an informed choice and are unlikely to respond to further interventions.

### Implications to practice and research

A Cochrane Systematic review published in 2012 [35], reported uptake rates for general health checks were between 50% and 90% with a median of 82%. Although the review focused on update data from trials which tend to be higher than in real world settings, the original (and perhaps ambitious) target rate of 75% for NHSHC uptake in England was based on economic modelling for cost-effectiveness. However, as NHSHC implementation embeds itself across England and providers innovate to meet local needs, uptake rates appear to be improving; currently 51.3% compared with a five year average 46.5% [36].

However, screening for cardiovascular risk fundamentally differs from screening for existing disorders such as cancer [37]. Cancer screening is based on the probability that a future event will occur, splitting the population into people who are likely or unlikely to have the existing condition, before then confirming or ruling out the condition on the basis of further diagnostic tests. In contrast, cardiovascular risk is continuously distributed in the population. While a small proportion of individuals shown to be at high risk will then have CVD confirmed upon subsequent diagnostic testing, the majority of individuals will simply be identified as being at low, medium, or high level of risk for a future cardiovascular event. Individuals at medium or high risk will be offered treatments to help reduce their risk of a future cardiovascular event. The treatment options fall broadly into pharmaceutical management and behavioural change (e.g. smoking cessation, increasing physical activity and weight management). However, lifestyle interventions implicitly assume the individual has placed themselves at higher risk through ‘bad habits’, thereby placing a moral responsibility on the individual to make healthier choices [13,21]. This was reflected by comments from non-attendees not wishing to be ‘told off’ by health professionals.

A recent systematic review of patient uptake and completion of cardiovascular lifestyle behavioural change identified emotions, psychological beliefs, information and communication needs, support from family and friends, transport and other costs as barriers [38]. The current study confirmed that misunderstanding of the NHSHC was prevalent among non-attendees. Indeed, patients believed that if they received monitoring for other non-cardiovascular chronic conditions or had their blood pressure or cholesterol recently measured, a preventative cardiovascular health check was unnecessary. Furthermore, many non-attendees simply did not view it as a priority. Unfortunately, barriers to preventative interventions appear to revolve around patients not believing they are at risk of developing the disorder and poor communication about the service offered [25]. Clearly, greater promotion is required to propel NHSHCs into the public arena, and to accentuate that they can identify individuals who may benefit from a preventative programme that offers lifestyle advice to reduce CVD risk, as opposed to, for example, a diagnostic screening programme for cancer. Patient ideas to increase uptake echoed those suggested by Cuijpers et al [25] such as mass media campaigns, use of the internet and providing NHSHCs within community settings such as the gym or chemist.

## Conclusions

For the NHSHC programme to achieve its cost-effectiveness targets more must be done to improve uptake. Although the latest guidance has downgraded the acceptable uptake threshold from 75% to 50% [39], considerable scope remains for rigorous qualitative investigation with diverse patient groups to better understand the barriers and motivators to NHSHC attendance. Our study highlights the need for greater clarity, but also brevity, of invitation materials and more creative advertising. Future studies should test the impact of simple interventions (e.g. systematic use of reminder letters). Our findings highlight the potential of opportunistic discussions between a health professional and patient, a method of proven effectiveness in improving cancer screening uptake rates. The NHSHC learning network has recently launched a greatly simplified invitation letter developed with support from the Department of Health Behavioural Insights team [40]. While our findings support the need for new materials, this may not be sufficient to tackle some of the primary barriers to attend i.e. that people fail to prioritise it over competing demands on their time, misunderstand its purpose or benefits, or make an informed choice not to go.

## Additional file

Additional file 1:
**Consolidated criteria for reporting qualitative studies (COREQ): 32-item checklist.**

## Abbreviations

CVD: Cardio-vascular disease
NHSHC: National Health Service cardiovascular health check
